# Unveiling the Complexities of Hereditary Angioedema

**DOI:** 10.3390/biom14101298

**Published:** 2024-10-14

**Authors:** Cristina Violeta Tutunaru, Oana Maria Ică, George G. Mitroi, Carmen Daniela Neagoe, George F. Mitroi, Olguța Anca Orzan, Beatrice Bălăceanu-Gurău, Simona Laura Ianoși

**Affiliations:** 1 Department of Dermatology, Faculty of Medicine, University of Medicine and Pharmacy of Craiova, 200349 Craiova, Romania; crisbej@yahoo.com (C.V.T.); oana.maria.corici@gmail.com (O.M.I.); simonaianosi@hotmail.com (S.L.I.); 2 Department of Internal Medicine, Faculty of Medicine, University of Medicine and Pharmacy of Craiova, 200349 Craiova, Romania; daniela.neagoe@umfcv.ro; 3 Department of Urology, Faculty of Medicine, University of Medicine and Pharmacy of Craiova, 200349 Craiova, Romania; george.mitroi@umfcv.ro; 4 Department of Oncologic Dermatology, “Elias” Emergency University Hospital, “Carol Davila” University of Medicine and Pharmacy, 020021 Bucharest, Romania; 5 Clinic of Dermatology, “Elias” Emergency University Hospital, 011461 Bucharest, Romania

**Keywords:** hereditary angioedema, biomarkers, genetics, C1-INH, FXII, SERPING1 gene

## Abstract

Hereditary angioedema (HAE) is a rare and potentially life-threatening genetic disorder, constituting approximately 2% of all clinical cases of angioedema, with a global prevalence estimated between 1 in 50,000 and 1 in 150,000 individuals. The condition affects individuals of all genders and ethnic backgrounds without significant variation. HAE is classified into three types. Type I HAE, which accounts for 85% of cases, is characterized by a deficiency of the C1 esterase inhibitor (C1-INH) gene. Type II HAE, making up 15% of cases, involves a dysfunctional C1-INH. Type III HAE, which represents about 5% to 10% of cases, is often estrogen-dependent and although several mutations have been identified, it typically involves normal C1-INH activity. Despite the differences in C1-INH functionality, all three types of HAE manifest with similar clinical symptoms. HAE leads to recurrent episodes of non-pruritic angioedema, which occurs in the absence of urticaria. Breakthroughs in understanding HAE pathophysiology have revolutionized treatment, leading to the development of highly targeted therapies for both acute management and long-term prevention. Meanwhile, cutting-edge advancements in omics technologies are unlocking new possibilities for biomarker discovery, paving the way for more precise diagnoses and personalized treatment strategies that could significantly enhance patient outcomes. This review will delve into the intricate pathophysiology, diverse clinical presentations, and diagnostic challenges of HAE while exploring emerging biomarkers and innovative approaches to therapeutic management and prevention strategies. Additionally, it will underscore the vital importance of screening family members of affected individuals, even when symptoms are not present.

## 1. Introduction

Hereditary angioedema (HAE) is a rare genetic disorder caused by either a deficiency or dysfunction of the C1 esterase inhibitor (C1-INH) gene, which underlies both type I and type II HAE, as well as the existence of hereditary angioedema with normal C1-INH activity (type III HAE) [1,2,3,4,5].

The Hereditary Angioedema Working Group (HAWK) classification has helped clarify the confusion that has surrounded this topic for years by distinguishing between hereditary and acquired forms of angioedema, regardless of whether they are associated with C1-INH deficiency [6]. Recent research has identified additional mechanisms in angioedema pathogenesis, including the unchecked activation of factor XII, the production of vasoactive mediators that disrupt endothelial function, and the interplay between mast cell-derived mediators and the plasma contact system [6].

The pathogenesis of HAE involves the accumulation of extravascular fluid in various tissues through a non-inflammatory and non-allergic mechanism [7]. The increase in vascular permeability that leads to angioedema in HAE is closely associated with the mediators of the contact system, specifically the kallikrein–kinin pathway, where C1-INH plays a critical regulatory role across several physiological pathways, including fibrinolysis, coagulation, the contact system, and the complement system [1,7]. Inhibition of plasma kallikrein and coagulation factor FXIIa by C1-INH is essential [7]. When C1-INH function is compromised, bradykinin overproduction occurs, which then activates B2 receptors, increasing vascular permeability and leading to angioedema [7,8,9]. As the pathophysiological mechanisms underlying HAE involve numerous molecules, they may serve as potential biomarkers [10].

There are three types of HAE, namely type I, II, and III, which are differentiated by etiology and blood levels of the C1 inhibitor [1,10]. HAE types I and II result from mutations in the SERPING1 gene, which encodes the synthesis of the C1-INH protein responsible for regulating inflammation [1,10]. Type III HAE, often referred to as estrogen-dependent HAE or HAE with normal C1-INH activity, typically occurs in individuals with normal C1-INH levels and is often influenced by hormonal factors such as estrogen [1].

Symptoms of HAE generally begin in childhood or young adulthood, often worsening around puberty [1]. Clinically, HAE is characterized by the sudden onset of swelling [7]. Angioedema, a hallmark of HAE, presents as non-pitting edema in subcutaneous and submucosal tissues, commonly affecting the lips, face, neck, extremities, oral cavity, and larynx [1,11]. It is important to acknowledge the possibility of genital and intestinal angioedema, as these sites are often overlooked in clinical assessments [1,11]. Patients typically experience recurrent episodes of swelling or abdominal pain and on occasion, a serpentine, non-itchy rash may appear [1,12,13,14,15,16]. Most acute episodes of HAE resolve within one to several days [7]. The most concerning complication is laryngeal edema, which can lead to breathing difficulties and asphyxiation, though such severe cases are relatively rare [17,18].

Recent advances in understanding the pathophysiology of HAE have led to the development of newer, targeted therapies for both acute management and long-term prevention. High-throughput omics-based technologies are also advancing rapidly, offering the potential for identifying new candidate biomarkers, which could significantly improve diagnosis and enable a more personalized approach to prevention and treatment [10]. However, keeping up with these evolving therapeutic options can be challenging for clinicians.

This review will explore the pathophysiology, clinical manifestations, and diagnosis of HAE, as well as discuss potential biomarkers, therapeutic management strategies, and prophylaxis options. Additionally, the review will emphasize the importance of screening family members of affected individuals, even if they are asymptomatic, to ensure early diagnosis and intervention.

## 2. Materials and Methods

**Figure 1:**
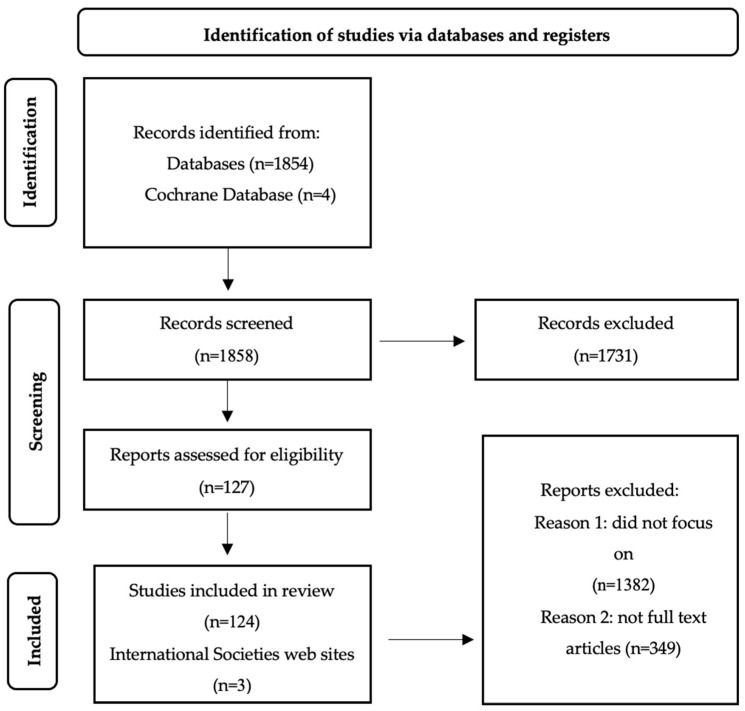
The flowchart underlines the studies included in our research.

We conducted online research using Web of Science Core Collection, Medline (PubMed), ResearchGate, and Cochrane electronic databases for review articles written in English from the last 10 years, using keywords such as “hereditary angioedema”, “bradykinin”, “C1 inhibitor protein”, and “SERPING 1 gene”. Articles were selected based on their relevance to our study, excluding those without direct relevance. From the initial 1854 articles reviewed, we focused our analysis on 130 review articles published in English over the past 10 years (Figure 1).

## 3. Discussions and Results

J. L. Milton first mentioned HAE in 1876 and in 1882, Quincke introduced the term “angioneurotic edema” to describe the condition [19,20,21]. The term “neurotic” was included because mental stress was observed to exacerbate the disease [19,21]. In 1888, Sir William Osler provided a comprehensive description of HAE across five generations, highlighting the hereditary nature of the condition [19,22]. The biochemical basis of hereditary angioneurotic edema—specifically, the deficiency of C1-INH—was uncovered by Donaldson and Evans in 1963 [19,23]. Since then, substantial progress has been made in understanding the genetic basis, pathophysiology, clinical manifestations, and management of HAE, leading to a wealth of published research on the topic [19].

### 3.1. Epidemiological Data

HAE affects approximately 1 in 50,000 individuals, constituting approximately 2% of all clinical cases of angioedema [1,3,4,5]. The condition affects individuals of all genders and ethnic backgrounds without significant variation [5].

Type I HAE represents about 80% to 85% of cases, while type II HAE accounts for 15% to 20% of cases and type III makes up approximately 5% to 10% of all HAE cases [19]. The condition typically manifests as cutaneous swelling in nearly 75% of patients, while approximately 25% experience severe abdominal attacks [19,24,25,26,27]. In one patient series, recurrent abdominal pain and facial or upper airway edema were reported in 52% and 36% of cases, respectively [19,28]. Additionally, 39% of patients identified a traumatic event as the trigger for their initial episode [19,28].

The onset of types I and II HAE occurs before the age of five in about 40% of individuals, with three-quarters experiencing symptoms by age 15 [29,30]. Patients may initially exhibit mild unnoticeable symptoms that escalate in severity around puberty [29,30]. In contrast, type III HAE typically arises during the second decade of life, often after puberty [29,30].

Although HAE persists throughout life, some patients may experience improvements with age [29,30]. Notably, 5% of adult carriers remain asymptomatic and are often not diagnosed until their children present with symptoms [29,30].

### 3.2. Etiopathogenesis

HAE is inherited in an autosomal-dominant manner, reflecting that children have a 50% chance of inheriting the condition if one parent is affected [1,31,32]. However, the absence of a family history does not rule out the diagnosis, as approximately one-quarter of cases arise from a spontaneous mutation in the C1-INH gene during conception [1,3,31,32]. The gene responsible for HAE is located on the long arm of chromosome 11 (11q12-q13.1) [6,8,31,32].

C1-INH is a 105 kDa α2-globulin that belongs to the serpin family of serine protease inhibitors, which also includes proteins such as alpha-trypsin, angiotensinogen, and antithrombin III [1,17,31,32,33]. These proteins are characterized by their ability to form stable one-to-one complexes with and inhibit their target proteases [1,6,9,17,31,32,33]. The C1-INH gene is primarily produced by hepatic cells, although peripheral blood monocytes and skin fibroblasts also contribute to its synthesis [1,3,17,31,32,33].

Cytokines play an important role in stimulating C1-INH synthesis [17,18,23,31,32,33]. Interferon-γ specifically enhances C1-INH production in vivo [17,18,23,31,32,33]. Interleukin-6 (IL-6) promotes the release of C1-INH from HepG2 liver cells, an effect that is further amplified by interleukin-1 (IL-1), which does not directly influence C1-INH production [17,18,23,31,32,33]. The regulation of C1-INH is complex and not fully understood, as some patients respond positively to androgen therapy despite having elevated serum levels of C1-INH [17,27,31,32,33]. It is hypothesized that androgens may enhance C1-INH synthesis and inhibit the activation of the lectin pathway by interacting with mannose-binding lectin associated with serine proteases (MASP) [3,17,31,32,33].

**Figure 2:**
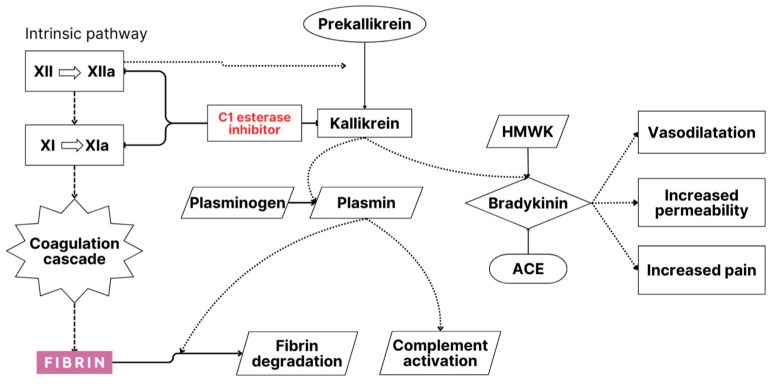
Graphical presentation explaining the possible sequence of events involved in the etiopathogenesis of HAE. Regular arrows indicate standard activation or progression pathways within the cascade. For example, the activation of Prekallikrein to Kallikrein and the conversion of Plasminogen to Plasmin show standard biochemical transitions. Dashed arrows represent indirect or regulatory relationships within the system. For instance, the dashed line from Kallikrein to the intrinsic pathway components, such as Factor XIIa, indicates an indirect or less immediate involvement in activating that process. Dotted arrows represent effects or consequences of certain reactions. For example, the dotted lines leading from Bradykinin to Vasodilatation, Increased Permeability, and Increased Pain illustrate the physiological outcomes mediated by bradykinin. Red Text (C1 Esterase Inhibitor): This highlights the role of the C1 Esterase Inhibitor, which is essential in controlling the activation of kallikrein and, therefore, the production of bradykinin. It’s shown in red to emphasize its importance as a therapeutic target or regulator in the pathway. Pink Text (Fibrin): This indicates the formation of Fibrin, a key component of the coagulation cascade, which links to the fibrinolysis process mediated by plasmin. It may be colored to emphasize the distinction between the clotting pathway and the bradykinin-mediated angioedema pathway. HMWK—high-molecular-weight kininogen; ACE—angiotensin-converting enzyme.

Bradykinin is the primary mediator implicated in HAE, and C1-INH plays a crucial role in regulating its production [1,3,31,32,33] (Figure 2). During traumatic or stressful events, the activation of the contact and complement pathways can occur, leading to elevated bradykinin levels [27,31,32]. Bradykinin, by binding to its receptors on endothelial cells, can increase vascular permeability, thereby contributing to the development of recurrent episodes of angioedema [27,31,32] (Figure 2).

C1-INH is essential for regulating several biological systems, including the complement cascade (specifically C1r, C1s, MASP-1, and MASP-2), the coagulation and contact protease systems (which involve plasma kallikrein, activated Hageman factor, and coagulation factors XIIa, XIIf, and XIa), and the fibrinolytic pathway via plasmin [1,8,9,31,32,33] (Figure 2). In the absence of C1-INH, the activation of C1, C2, and C4 proceeds unchecked, potentially overwhelming other regulatory inhibitors like C4-binding protein, factor H, and factor I [30]. This disruption can lead to a dysregulated cascade, resulting in impaired control of the complement system (Figure 2).

The complement system comprises nine components (C1–C9) and operates through two primary pathways of activation, namely the classical and alternative pathways [18,23,32,33] (Figure 2).

C1 is a heterotetrameric complex, consisting of one C1q subunit, two C1r subunits, and two C1s subunits, all interconnected by calcium ions [17,32,33]. In the classical pathway, complement activation is initiated when the Fab fragment of an immunoglobulin binds to its target antigen [1,3,17,33]. This interaction facilitates the engagement of C1q with the Fc region of the antibody [1,3,17,33]. As a result, C1r and C1s are recruited, leading to the activation of C1s, which acquires esterase activity [1,3,17,33]. This binding recruits C1r and C1s, resulting in the activation of C1s, which gains esterase activity [1,3,17,33]. Once activated, C1s cleaves C4, triggering a cascade that produces various complement fragments, including the membrane attack complex, which is responsible for lysing target cells [1,3,17,33]. During this process, fragments such as C3a, C4a, and C5a are produced, contributing to increased vascular permeability and edema in tissues, which are characteristic of an HAE attack [1,3,17,33].

Normally, when there is an insufficient amount of C1-INH, circulating C1 can become hyperactivated [17,18,23,33]. C1-INH functions to prevent this by dissociating the C1q subunit from the complex, resulting in the formation of an inactive C1r2-C1s2-(C1-INH)2 complex [6,8,17,33]. This complex is unable to activate the complement components C4 and C2, thereby blocking the activation of the classical complement pathway and preventing excessive immune responses [6,8,17,33].

Signs and symptoms are similar in all types of HAE [29,30].

Type I HAE is the most prevalent form of the disorder, resulting from a deficiency in the C1-INH protein due to various genetic mutations [1,29,30]. These mutations, including misdirection, deletions, or insertions, lead to the production of truncated or misfolded C1-INH proteins [1,29,30]. Consequently, affected individuals exhibit reduced antigenic and functional levels of C1-INH, with plasma concentrations typically falling between 5% and 30% of normal levels [1,29,30]. Even in patients possessing one normal allele, the overall production of functional C1-INH is insufficient, potentially due to the downregulation of C1-INH levels, as indicated by low levels of C1-INH mRNA in affected individuals [1,29,30]. Additionally, C1-INH may bind to target proteins, leading to its inactivation and subsequent clearance from the bloodstream [1,29,30].

Type II HAE is distinguished from type I HAE by the presence of normal or even elevated levels of C1-INH protein, but with impaired functional activity [1,3,29]. The dysfunction in type II HAE arises from mutations in one allele of the gene responsible for C1-INH production, while the other allele remains normal [1,29,30]. Type II HAE is characterized by high allelic heterogeneity, with approximately 748 documented mutations [1,27,29]. These mutations lead to low levels of functional C1-INH protein despite normal or increased antigenic levels of the mutant protein [1,29,30]. The deficiency of functional C1-INH allows for the uncontrolled autoactivation of C1, resulting in the consumption of complement components C4 and C2 [1,9,29,30].

The proper function of C1-INH requires an intact peptide between amino acids [1,6,29,30]. The mutations often occur in the reactive center of C1-INH, particularly at the Arg444-Thr445 site, which is essential for binding and inhibiting target proteases [1,29,30]. Mutations that substitute arginine at position 444 with cysteine or histidine are involved in up to 70% of type II HAE cases, resulting in the production of C1-INH that is present, but lacks functional activity [27,29,30]. Additionally, a significant proportion of type II HAE cases are caused by mutations in the reactive center loop (RCL) of the C1-INH protein, with some mutations affecting the Lys251 amino acid, which disrupts proper protein folding and function [1,29,30].

As previously mentioned, both type I and type II HAE are caused by mutations in the SERPING1 gene, which encodes the C1-INH protein [1,29,30]. According to Bork et al. (2018), approximately 300 different genetic mutations can lead to HAE, with about 25% of these mutations occurring spontaneously [34].

Type III HAE, a much rarer form, often referred to as estrogen-dependent HAE or HAE with normal C1-INH activity, typically occurs in individuals with normal C1-INH levels [1]. It is often influenced by hormonal factors such as estrogen [1]. Unlike types I and II, type III HAE is not directly associated with C1-INH deficiency, but is linked to other genetic mutations, particularly in the kininogen-1 gene (HAE-KNG1), plasminogen gene (PLG-HAE), myoferlin gene mutation (MYOF-HAE), heparan sulfate-glucosamine 3-sulfotransferase 6 (HS3ST6), mutation in Hageman factor (factor XII), and in the angiopoietin-1 (HAE-ANGPT-1) gene [1,18,23,29,30]. These mutations affect the kallikrein–kinin and fibrinolytic system pathways, leading to increased bradykinin production and consequent vascular permeability, resulting in angioedema [1,29,30]. Current guidelines now recommend subdividing HAE with a normal C1-INH gene (HAE-nl-C1-INH, formerly known as type III HAE) based on the underlying mutations [1,18,35].

Type III HAE is associated with an increased activity of kininogens, leading to elevated bradykinin levels [1,29,30]. This rise in bradykinin may be attributable to an inherited deficiency or functional decrease in enzymes responsible for its degradation, such as angiotensin-converting enzyme (ACE), carboxypeptidase N, and α2-macroglobulin [1,29,30]. Another possibility is the production of an unidentified substance that, independent of C1-INH regulation, generates bradykinin by cleaving high-molecular-weight kininogen [1,29,30].

Type III HAE has often been described as estrogen-dependent because it primarily affects women and is exacerbated by high estrogen levels [1,3,29,30]. However, the exact mechanism by which estrogen influences angioedema is not fully understood, and the term “estrogen-dependent HAE” is somewhat misleading [1,8,29,30]. It is hypothesized that estrogen may upregulate bradykinin production and simultaneously inhibit its degradation through ACE, thereby contributing to the angioedema observed in type III HAE [1,29,30].

Although C1-INH levels are normal in type III HAE, the physiological defect that causes angioedema may be related to decreased kallikrein activity [29,30]. Kallikrein, along with factors XIIa and XIIf, is normally inhibited by C1-INH [1,8,9,29,30]. Consequently, in type III HAE, the regulation of kallikrein and related pathways may be disrupted, leading to excessive bradykinin production and subsequent episodes of angioedema [1,8,29,30].

### 3.3. Signs and Symptoms

HAE symptoms typically manifest in childhood, with approximately 50% of individuals becoming symptomatic by age seven and 66% by age thirteen [35,36]. In childhood, attacks are generally mild, infrequent, and predominantly present as abdominal pain, but symptoms progressively worsen during puberty [35,36]. In addition, about one-third of affected individuals may experience prodromal symptoms, such as paresthesia, sudden mood changes, sensory alterations, anxiety, or fatigue, before an attack [30,34]. Some may also present with erythema marginatum, a flat, non-pruritic erythematous rash that can resemble urticaria, although it is more commonly associated with rheumatic fever [30,34]. Severe cases can lead to the development of bullae or blisters [30,34].

Attacks are often triggered by various factors, although only around 40% of patients can identify a specific one [1,24,36,37]. Common triggers include physical injury, intense pain, stress, anxiety, surgical procedures (such as dental work), viral infections, and certain physical activities [1,24,36,37]. Importantly, ACE inhibitors can increase the frequency and severity of HAE attacks and should therefore be avoided [1,24,36,37].

HAE symptoms can vary widely among individuals and include angioedema, abdominal pain, ascites, and intestinal edema [1,6,7]. Attacks typically occur in one area but also can present in various areas, including subcutaneous tissue (hands, legs, arms, genitalia, and buttocks), abdominal organs (stomach, intestines, gallbladder, and kidneys), and the upper respiratory tract (larynx and tongue) [1,6,7]. Individuals with normal C1-INH levels are particularly prone to facial edema [1,6,7]. Cutaneous urticaria is rarely observed [1,6,7].

The hallmark symptom of HAE is non-inflammatory cutaneous and mucosal edema, primarily affecting the extremities, genital area, and face [1,24,36,37]. This edema usually worsens within 12–24 h and can persist for up to 5 days, with potential migration to other areas [1,24,36,37]. The condition is characterized by a sensation of constriction or paresthesia before the edema fully develops and is unresponsive to antihistamines, corticosteroids, epinephrine, and anti-IgE (omalizumab) [1,36,37,38]. Attacks are recurrent, with symptom-free intervals often lasting several weeks, and can progressively worsen [1,24,36,37].

Digestive system edema can cause acute abdominal pain, with ascites often present during abdominal attacks, causing nausea, vomiting, and signs of obstruction such as dehydration [38]. Diarrhea or constipation may also occur, with symptoms usually resolving within 12–24 h [38].

Pharyngeal or laryngeal edema can lead to a range of serious symptoms, including coughing, dysphagia, dysphonia, stridor, and asphyxiation [39]. About half of patients experience at least one laryngeal attack, which typically begins with alterations in voice and swallowing difficulties [39]. These laryngeal attacks are particularly concerning, as they account for approximately 30% of deaths related to HAE, with a 70% risk of patients experiencing such an attack [39]. They can be triggered by local anesthetics used during dental procedures, although they may also occur spontaneously [39].

Other reported symptoms are mentioned in Table 1 [40,41,42].

In women, hormonal changes, particularly during menstruation, can influence HAE symptoms, with some experiencing a higher frequency of attacks during this period [1,36,37]. Estrogen-based medications, such as oral contraceptives and hormone replacement therapies, have been associated with an increased frequency and severity of HAE attacks [1,24,36]. Despite this, specific triggers often remain unidentified [1,24,36].

The scientific literature shows significant variability in attack frequency among pregnant women [1,36,37]. Pregnancy is generally associated with increased serum C1-INH levels, which may reduce the risk of attacks rather than exacerbate them [1,36,37]. This protective effect is likely due to the overall increase in circulating C1-INH during pregnancy, although a relative decrease in C1-INH levels may occur due to significant plasma volume expansion, particularly in the last trimester [1,24,36,37]. C1-INH levels are also observed to be low in pregnant women with pre-eclampsia and eclampsia [1,36,37]. A significant proportion of pregnant women with HAE have been reported to experience premature births [1,36,37].

Patients with HAE may have an increased susceptibility to autoimmune diseases such as inflammatory bowel disease, systemic lupus erythematosus (affecting approximately 2% of patients), thyroiditis, Sjögren’s syndrome, drug-induced lupus, pernicious anemia, scleroderma, and autoimmune aortitis [2,40,41]. Although patients may experience malaise, they typically remain afebrile [2,40,41]. Ensuring a clear airway is crucial, especially in severe cases where seizures might lead to hypotension due to fluid sequestration [2,40,41].

Untreated individuals typically experience attacks every 1–2 weeks, with each episode lasting 3–4 days [35,36]. The frequency and duration of attacks can vary significantly among patients and within the same family [35,36].

### 3.4. Diagnosis

#### 3.4.1. Laboratory Testing

HAE diagnosis is based on a thorough clinical evaluation, family history, and specific blood tests to detect a deficiency in C1-INH, which leads to elevated bradykinin levels [1,24]. Understanding the distinct laboratory profiles is essential for accurate diagnosis and effective management [1,24].

Types I and II HAE should be suspected in patients with a history of recurrent skin swelling (affecting extremities, face, and genitalia), gastrointestinal attacks (painful abdominal cramps), and/or laryngeal edema [18,42,43,44]. The suspicion increases if the patient also reports any of the following: a positive family history; onset of symptoms during childhood or adolescence; recurrent and painful abdominal symptoms; episodes of upper airway edema; failure to respond to antihistamines, glucocorticoids, omalizumab, or epinephrine; prodromal signs or symptoms preceding swellings; and the absence of wheals [18,42,43,44].

**Figure 3:**
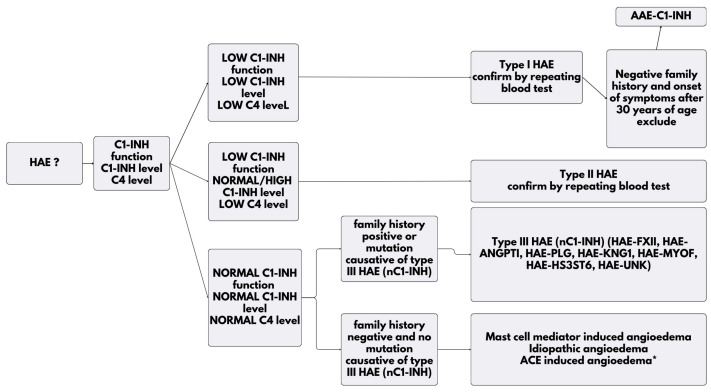
Diagnostic algorithm of HAE. The diagnostic evaluation for hereditary angioedema (HAE) involves distinguishing between various subtypes of the condition. Type I HAE is caused by a deficiency of the C1 inhibitor (C1-INH), while type 2 HAE stems from dysfunctional C1-INH. Acquired angioedema (AAE-C1-INH) occurs due to C1-INH deficiency that develops later in life, often related to other diseases. Hereditary angioedema with normal C1-INH levels (nC1-INH) is associated with mutations in genes such as ANGPT1, PLG, KNG1, MYOF, and HS3ST6, or remains genetically unexplained (labeled as unknown or UNK). * Additionally, certain drugs, including ACE inhibitors, angiotensin II receptor blockers, gliptins, neprilysin inhibitors, and tissue plasminogen activators, can induce bradykinin-mediated angioedema.

When types I and II HAE are suspected, laboratory investigations are recommended to confirm the diagnosis [18,45,46,47,48,49] (Figure 3). The primary diagnostic tests include measurements of serum or plasma levels of C1-INH function, C1-INH protein, and C4 [18,45,46,47,48,49] (Figure 3). The combined use of these three tests provides high diagnostic accuracy, greater than relying on any single test alone [18,45,46,47,48,49]. If a patient presents with low circulating C1-INH levels, they are likely to have type I HAE, whereas normal circulating C1-INH levels with dysfunctional protein suggest type II HAE [18,45,46,47,48,49] (Table 1).

C1-INH deficiencies impair the complement cascade, leading to low functional levels of C4 during an attack and in intervals between frequent episodes [1,24,40,50]. If angioedema is suspected but the C4 level is normal, the test should be repeated [1,24,40,50]. Occasionally, C4 levels remain normal at the onset of an attack [1,24,40,50]. Random C4 level checks have a sensitivity of about 80%, which can be improved by drawing blood during an emerging attack, though this does not increase specificity [1,18,24,45,46,47,48,49,50]. Monitoring D-dimer levels may also aid in diagnosing an acute HAE attack, as D-dimer levels tend to rise in this context [1,24,40,50]. If any of these lab values are 50% or less than normal, the tests should be repeated within 1–3 months to confirm accuracy and rule out an acute illness as the cause of the abnormal results [1,24,40,50].

For a definitive diagnosis of HAE, it is recommended that patients exhibit both clinical symptoms and corresponding positive laboratory findings [1,24,40,50] (Figure 2).

For type III HAE with normal C1 inhibitor levels, diagnosis is based on a history of recurrent angioedema without urticaria or causative medication; normal or near-normal levels of C4, as well as antigenic and functional C1-INH; the presence of a genetic mutation defects in one of the six genes as follows: FXII, PLG, ANGPT1, KNG1, MYOF, and HS3ST6; a positive family history of angioedema, or a lack of efficacy of high-dose antihistamines (e.g., cetirizine 40 mg/day for at least one month or during three angioedema attacks) [7,51,52]. Genetic testing is indicated if there are recurrent episodes of angioedema of unknown etiology [7,51,52] (Figure 3). However, genetic testing has limitations, particularly in predicting disease progression. A positive genetic result does not reliably predict the severity or specific symptoms, as the same genetic variation can manifest differently among individuals.

In cases of type III HAE, the levels of C4 remain normal [7,51,52] (Table 1).

HAE patients typically have normal levels of C3 and C1q, regardless of disease status [1]. Acquired angioedema linked to lymphoproliferative diseases is often associated with low C1q levels [1]. Serum total hemolytic complement (CH50) is usually low during attacks but returns to normal afterward; however, this test is less useful, as any complement deficiency can lower CH50 [1]. Although patients with HAE usually present normal results in standard tests, some may show hemoconcentration or prerenal azotemia during attacks, reflecting intravascular volume loss [7,51,52]. Leukocyte counts typically remain normal, though they may increase during abdominal attacks [7,51,52].

It is important to note that the availability and quality of the laboratory tests can vary globally [18,45,46,47,48,49]. In some regions, the diagnostic approach may need to be adapted based on the local availability of these tests and guidelines should be used to advocate for the necessary diagnostic resources in order to reduce the mortality and morbidity associated with HAE (Table 2) [18,45,46,47,48,49].

#### 3.4.2. Imaging Studies

Imaging studies can occasionally aid in diagnosing angioedema [2]. An abdominal X-ray may reveal signs of ileus during gastrointestinal angioedema [2]. A chest X-ray, though less common, might detect pleural effusions [2]. Abdominal ultrasound or computed tomography (CT) scans can show a thickened bowel wall due to edema, fluid accumulation around the bowel, and in some cases, significant free peritoneal fluid [2].

#### 3.4.3. Histology

Histologically, HAE is characterized by a perivascular mononuclear infiltrate and dermal edema, similar to those seen in chronic urticaria or other angioedema types [2]. Edema is observed in the reticular, subcutaneous, or submucosal dermis without an inflammatory cellular infiltrate [2]. Vasodilation may be present [2].

### 3.5. Biomarkers

The pathophysiologic pathways in HAE involve numerous molecules from the complement, coagulation, and fibrinolysis systems, as well as from the vascular endothelium [10]. These molecules have the potential to serve as biomarkers for the condition [10] (Table 3). For these biomarkers to be highly specific, they must be closely associated with the pathological mechanisms of HAE, particularly the bradykinin-generating cascade [10].

Antigenic C1-INH (AgC1-INH) plasma concentration is an essential diagnostic biomarker for identifying type I HAE [10,33]. Spath et al. demonstrated that the frequency of attacks was highest in patients with HAE when AgC1-INH levels were below 0.035 g/L [98]. Nevertheless, other studies have reported that AgC1-INH levels tend to be lower during attacks or show a negative correlation with the annual number of attacks [53,70].

The protease–inhibitor complex C1-INH-C1(r,s) is indicative of contact system activation and may be considered a potential biomarker for HAE [10,54]. Studies have shown that plasma levels of C1-INH-C1(r,s) complexes are higher in patients with HAE compared to healthy controls [53,55,56]. Moreover, plasma levels of C1-INH-C1(r,s) complexes further increase during angioedema attacks [53,55,56]. Patients with elevated levels of C1-INH-C1(r,s) complexes have a history of more severe attacks and require more frequent emergency treatment [10,56]. Moreover, plasma C1-INH-C1(r,s) complex levels have been reported to normalize in patients treated with stanozolol, accompanied by a reduction in symptoms, suggesting its potential as a biomarker for monitoring therapeutic response [10,57].

Functional C1-INH (fC1-INH) plasma levels, also known as C1-INH activity, are a key diagnostic test for identifying type II HAE [47,54,58,59]. Research by Kelemen et al. found that baseline fC1-INH levels correlate with the severity of HAE [60]. Further studies by the same group revealed that patients with lower fC1-INH levels experienced more recurrent attacks and a greater need for on-demand treatment [53,60]. A functional C1-INH level around 40% seems to provide sufficient protection against angioedema episodes for most patients undergoing prophylactic subcutaneous C1-INH therapy [61,62,63]. This aligns with previous clinical findings, where maintaining this threshold was found to significantly reduce the frequency and severity of attacks [61,62,63]. Therefore, fC1-INH levels within this range may serve as a prognostic biomarker for assessing the likelihood of future attacks [10].

Complement C4 is a significant diagnostic biomarker in HAE, as its levels are typically reduced in most patients, particularly during attacks [54,61]. Research has shown that complement C4 levels correlate with the frequency of attacks and the on-demand use of C1-INH concentrate, though they do not correlate with disease severity scores [53,60].

Varga et al. discovered that anti-C1-INH IgM antibody levels are linked to the disease’s severity in patients who have not been treated with C1-INH concentrate [64,65]. Additionally, other studies have shown that the levels of MASP-1 and MASP-2-C1-INH complexes are lower in patients with HAE and are related to the frequency of attacks [65,66,67]. Conversely, the levels of MASP-2 and ficolin-3/MASP-2 complexes increase during angioedema attacks [65,66,67].

Since bradykinin is the primary mediator of swellings in HAE, it is anticipated to be the most precise biomarker for predicting upcoming attacks [10,57,63]. Plasma bradykinin levels have been reported to be higher in patients with HAE compared to healthy controls, with a further significant increase during attacks [10,68,69]. Additionally, a study by Nussberger et al. found higher bradykinin levels in blood samples taken from the site of angioedema compared to the control sites [10,68,69]. However, the clinical utility of plasma bradykinin levels is under scrutiny due to their high sensitivity to pre-analytical procedures and their extremely short half-life, which is measured in seconds [10,54,60].

High-molecular-weight kininogen (HK) undergoes proteolysis by active plasma kallikrein (PKa), leading to the generation of cleaved HK (cHK) and bradykinin [10]. As a result, cHK is considered a promising indirect marker of bradykinin release and contact system activation, which are key processes during HAE attacks [10]. Suffritti et al. reported that cHK levels are elevated in patients with HAE compared to controls and these levels further increase during attacks [10,70]. Additionally, they have demonstrated that cHK levels are higher in highly symptomatic patients compared to those with less frequent attacks [10,70]. Consequently, cHK levels can effectively differentiate between patients and healthy individuals, distinguish between those experiencing severe and mild symptoms, and determine whether a patient is undergoing an acute attack or is in a state of remission [10]. Developing a new, reliable, and less labor-intensive measurement method could enhance the clinical use of this parameter [10].

Plasma kallikrein (PKa) is also regarded as a potential biomarker for bradykinin-mediated angioedema attacks [10]. Measurements of spontaneous PKa activity using a chromogenic substrate have revealed elevated levels in patients with HAE compared to controls, with a further increase observed during attacks [70].

Activated coagulation factor FXII (FXIIa) has been identified as a potential biomarker for type III HAE [10]. Studies have shown that FXIIa levels are elevated in patients with C1-INH-HAE compared to healthy controls, and these levels increase further during attacks [71,72,73]. However, the utility of FXIIa as a biomarker for FXII-HAE during symptom-free periods remains uncertain given the variability in research findings [99,100]. Some studies have observed increased FXIIa activity in symptom-free patients, while others have found no significant difference between patients and healthy controls [99,100].

Konings et al. investigated factor XIIa/C1-INH complexes and found that levels of these complexes, along with FXIa-C1-INH and PKa-C1-INH complexes, were lower in patients with HAE compared to healthy controls, following the in vitro activation of samples with an FXII trigger [101].

The degradation and accumulation of kinins, including bradykinin, can influence the clinical presentation of HAE [10,74]. Major enzymes involved in kinin catabolism include carboxypeptidase N (CPN), ACE, and aminopeptidase P (APP) [10,74]. In FXII-HAE patients, disease severity was inversely related to both ACE and CPN activities, though this was not the case for APP [10,74,75,76]. Additionally, the total activity of serine proteases was found to be elevated in patients with types I, II, and III HAE compared to healthy controls, and it was further heightened in patients during attacks in type III HAE [76].

Plasminogen activator inhibitor (PAI)-1 levels, prothrombin time, and activated partial thromboplastin time (aPTT) were observed to be lower during type I and II HAE attacks compared to symptom-free periods [73]. Although PAI-1 levels were also lower in patients with FXII-HAE compared to controls, the difference was not statistically significant [102].

D-dimer levels were found to be elevated during HAE attacks and may help differentiate between abdominal HAE attacks and abdominal colic episodes, between multiple-site and single-site attacks, and between submucosal (e.g., abdominal, oropharyngeal–laryngeal) and subcutaneous (e.g., peripheral, facial) attacks [60,85,87]. Additionally, D-dimer levels tend to decrease approximately seven days after an attack [77,78].

The endothelium’s role in microvascular permeability and swelling in HAE has made it a key area for investigating potential biomarkers [10]. Research has explored several markers related to endothelial function and damage, including vascular endothelial cadherin (a transmembrane adhesive protein) (VE cadherin), von Willebrand factor (an indicator of endothelial damage) (VWF), soluble E-selectin (an adhesion molecule induced by cytokines), and endothelin-1 (a regulator of vasomotor activity) [77,79,80,81,82,83,84]. Other markers under investigation include arginine vasopressin, adrenomedullin, atrial natriuretic peptide, endothelial-derived endocan, and vascular cell adhesion molecule-1 (VCAM) (both markers of endothelial function) [10,79,80,81,82,83,84]. Further studies have examined vascular permeability modulators such as vascular endothelial growth factors (VEGFs), angiopoietin-1 (which stabilizes endothelial cells), and angiopoietin-2 (which enhances vascular permeability), as well as secreted phospholipases A2 (notably the 2A group) and platelet-activating factor acetylhydrolase (PAF-AH) [85,86,87,88]. Bova et al. found increased levels of angiopoietin-1 and VEGFs A and C in patients with unknown HAE, and elevated levels of VEGF C in patients with FXII-HAE [103].

Bas et al. explored 6-keto-prostaglandin F1-α, a long-lived metabolite of prostacyclin, as a potential biomarker for diagnosing angioedema induced by ACE inhibitors [10,95]. Additionally, Demirturk et al. found that plasma levels of endothelial nitric oxide (NO) synthase were significantly higher in patients with HAE in remission or during attacks compared to healthy controls [10,93]. Elevated levels of NO metabolites were observed only during attacks [10,93]. Further investigations have also focused on endothelial function, specifically examining asymmetric dimethylarginine (ADMA), a potent inhibitor of NO synthesis that is linked to various conditions like atherosclerosis [10].

Several studies have explored the relationship between HAE and markers of low-grade inflammation, elements of the immune system, and hormones. Researchers have compared patients with HAE to healthy controls using various parameters, including C-reactive protein (CRP), erythrocyte sedimentation rate (ESR), and white blood cell and neutrophil counts (WBCs) [89,90,91,92]. They have also investigated a broad array of cytokines, both pro-inflammatory and anti-inflammatory, such as IL-1β, IL-2, IL-4, IL-5, IL-6, IL-8, IL-10, IL-13, IL-17, interferon-γ, tumor necrosis factor-α (TNF-α), granulocyte colony-stimulating factor (G-CSF), and granulocyte macrophage colony-stimulating factor [92,93,94]. Additionally, the role of sex hormones, such as progesterone and sex hormone-binding globulin, has been examined [10].

Genomic biomarkers encompass DNA sequence variations—such as single-nucleotide variants, insertions, and deletions—as well as RNA alterations like differential gene expression and microRNAs [10,96,97]. In the context of angioedema, genetic testing focuses on identifying changes in genes involved in complement, fibrinolysis, coagulation, kinin, and vascular systems [10,96,97]. Key genes include C1-INH (SERPING1), FXII, PLG, and ANGPT1 [10,96,97]. While genomic biomarkers are primarily used to support the diagnostic process for HAE, research is ongoing to link genetic variations with disease severity and treatment responses, aiming to develop prognostic and predictive biomarkers [10,96,97].

Biomarkers have the potential to enhance diagnostic accuracy, enable personalized management, and support clinical trials in HAE [10]. Emerging technologies and advanced laboratory tests may help in establishing reliable biomarkers, but there is a pressing need for more well-designed studies and the completion of ongoing research to provide robust evidence of their effectiveness and utility. However, the advent of novel high-throughput techniques now allows for non-targeted multi-omics analyses (e.g., genomic, transcriptomic, proteomic, or metabolomic), enabling the identification of a cohort of biomarkers for further in-depth research [10].

### 3.6. Differential Diagnosis

There are several key conditions to consider in the differential diagnosis of HAE.

Acquired angioedema, which occurs without urticaria, is characterized by acute, non-inherited swelling affecting the skin and mucous membranes [1,41]. It can be triggered by allergens and is often treated similarly to urticaria [1,41]. In some cases, the pathophysiology resembles HAE and usually resolves within 1–2 days [1,41]. Common triggers include drugs, insect stings, and foods such as nuts, seafood, and eggs [1,41]. Severe allergic reactions can lead to angioedema [1,41].

Acquired angioedema is classified into two types [1,41]. Type I is associated with rheumatologic and hematologic conditions, including B-cell lymphoproliferative diseases (like chronic lymphocytic leukemia and multiple myeloma), macroglobulinemia, and essential cryoglobulinemia [1,41]. It can also result from conditions such as lupus anticoagulant, Churg–Strauss vasculitis, chronic infections (e.g., human immunodeficiency virus, hepatitis), or autoimmune diseases. This form is linked to circulating anti-idiotypic antibodies against B-cell surface immunoglobulins [1,41]. Other instances of acquired angioedema may arise from surgeries or malignancies [1,9,18,41]. It commonly presents after the fourth decade of life, whereas HAE typically manifests by the second decade [1,9,18,41]. Both conditions exhibit low C1-INH activity and similar clinical features, but they differ in underlying mechanisms [1,9,18,41].

Type II-acquired angioedema is often due to the production of an autoantibody that inhibits C1-INH function, the overuse of normal C1-INH, or factors from lymphoid tumors that disrupt C1-INH activity [1,9,18,41]. It is often associated with monoclonal gammopathy [1,9,18,41]. Unlike HAE, which maintains normal C1q levels, acquired angioedema usually shows decreased C1q levels [1,9,18,41].

HAE treatment differs from that of acquired angioedema; drugs used for the latter are ineffective against HAE [9,18,41]. When angioedema lacks urticaria or fails to respond to standard treatments, HAE should be considered [9,18,41].

ACE inhibitor-induced angioedema, though less common (less than 1% incidence), is more frequent in individuals of African ancestry (2.8–6%) [1,9,18,41]. It is influenced by factors such as smoking, older age, and gender [1,9,18,41]. This type of angioedema typically appears within the first week of ACE inhibitor use, but can also occur after prolonged use [1,9,18,41]. It generally resolves upon discontinuation of the medication and reappears with re-exposure [1,9,18,41]. ACE inhibitors are more likely to cause angioedema compared to angiotensin II receptor antagonists, and while NSAIDs rarely induce angioedema, it can occasionally occur a few hours after ingestion [1,9,18,41].

Histamine-mediated angioedema, often accompanied by urticaria and itching, can be triggered by factors such as viruses, medications, and certain foods [1,18,41]. Chronic cases typically involve either external agents or autoimmune conditions [1,18,41]. The release of histamine, which is controlled by mast cells via IgE or non-IgE pathways (e.g., due to opioids, contrast agents, or physical triggers), differentiates it from bradykinin-mediated angioedema [1,18,41]. Unlike histamine-mediated angioedema, bradykinin-induced cases often present with abdominal symptoms and do not respond to antihistamines [1,18,41].

Other different types of urticaria may also be taken into consideration, such as cholinergic, chronic spontaneous with angioedema, contact urticaria, vasculitis, dermographism, solar, and pressure urticaria [1,18,41]. Chronic spontaneous urticaria with angioedema is characterized by recurrent hives and swelling without an identifiable external trigger and is responsive to antihistamines [1,18,41]. Immediate hypersensitivity reactions (IgE-mediated) should also be considered as a differential diagnosis [1,18,41].

Additional types of angioedema include episodic angioedema with eosinophilia, pressure-induced angioedema, and those linked to rheumatological diseases, particularly if the edema is periarticular and restricts mobility [18,41]. Pressure or vibration-induced angioedema is triggered by physical pressure or vibration on the skin, leading to localized swelling [18,41]. Eosinophilia-associated angioedema is associated with elevated eosinophil counts and may be associated with swelling [18,41]. Vascular endothelial growth factor (VEGF)-related conditions, in which abnormalities in VEGFs can impact vascular permeability, can cause symptoms similar to angioedema [18,41].

Cutis laxa, a rare connective tissue disorder, presents with skin laxity, thickening, and hyperpigmentation [1,9,18,41]. Often diagnosed at birth or early childhood, its initial symptom is typically facial edema, which can be mistaken for HAE [1,9,18,41]. As cutis laxa progresses, it leads to notable changes in the skin and blood vessels [1,9,18,41].

Drug eruptions may cause swelling and rashes similar to HAE [1,9,18,41].

Various systemic conditions can also contribute to the development of angioedema. Systemic lupus erythematosus may cause angioedema alongside other systemic symptoms [1,9,18,41]. Dermatomyositis is characterized by skin manifestations and muscle weakness, often presenting with swelling [1,9,18,41]. Cutaneous Crohn’s disease can lead to swelling and skin lesions [1,9,18,41]. Additionally, facial cellulitis, caused by bacterial infection, results in redness, swelling, and warmth of the facial skin [1,9,18,41].

### 3.7. Prognosis

Overall, while HAE remains a serious condition with potential complications, the availability of effective treatments has markedly improved the prognosis and quality of life for many patients. Regular follow-up with a specialized healthcare provider and adherence to treatment plans are key to managing the condition effectively. Patients who are diagnosed early and receive appropriate therapy tend to have better control of their symptoms and a better overall outcome.

Patients with early-onset HAE often experience a more severe progression of the disease compared to those with later-onset attacks [1,2,104]. Historically, the mortality rate for HAE was 20–30% before the advent of effective treatments [1,2,104]. However, with appropriate prophylactic measures, the prognosis has significantly improved [1,2,104].

Although HAE is relatively rare, it can have severe consequences. Laryngeal edema may cause asphyxiation, requiring emergency interventions, and it may benefit from the self-injection of medication or emergency plans [1,2].

Abdominal attacks can result in intense pain, potentially leading to unnecessary medical interventions, diagnostic delays, and opioid dependence [1,2]. Subcutaneous HAE attacks can lead to disfigurement and disability, significantly impacting the patient’s quality of life [1,2]. Proper management and preventive treatment can help minimize these attacks [1,2].

The psychological and social impacts of living with a chronic condition like HAE can be significant [1,2]. Support from healthcare providers, counseling, and support groups can play a key role in managing the condition [1,2].

### 3.8. Standard Treatment

HAE is a multifaceted disorder that requires management by a multidisciplinary team. The pathophysiology of angioedema and increased vascular permeability in HAE is primarily mediated by bradykinin rather than histamine, rendering traditional treatments such as epinephrine, antihistamines, and glucocorticoids ineffective [18,105]. Consequently, the involvement of healthcare providers, patient counseling, and support groups is essential in the management of this condition [18,105]. Furthermore, it is critical to discontinue the use of ACE inhibitors, as they can elevate bradykinin levels and exacerbate angioedema episodes [18,105,106,107,108,109,110].

Pharmacological management is based on three key areas, namely the treatment of acute angioedema attacks (on-demand), short-term (preprocedural) prophylaxis, and long-term prophylaxis [18,105,106,107,108] (Table 4).

In the context of acute attacks, swelling typically resolves within 3 to 5 days without treatment [1,37]. The primary goal of acute treatment is to prevent rapid progression to severe complications, such as laryngeal edema and intense gastrointestinal pain, since peak symptom severity generally occurs within hours [1,39]. The early recognition of symptoms and the initiation of treatment within 6 h of onset have been associated with more favorable outcomes compared to delayed intervention [1,18,109].

Acute treatment options include plasma-derived C1-INH (pdC1-INH), recombinant human C1-INH (rhC1-INH), ecallantide, and icatibant [1,111] (Table 4). Patients should be properly educated on self-administration techniques, and it is recommended that they have two doses available at home to improve disease management [1,109]. A subcutaneous administration option can alleviate many challenges associated with self-administering IV formulations at home [1,111]. These medications typically become effective within 60 min, with relief often occurring within 2 h [1,110,111]. A second dose may be necessary if symptoms worsen [1,110].

If on-demand medications are unavailable, fresh frozen plasma (FFP) containing C1-INH can be used as an alternative, though it is not recommended due to the low level of evidence supporting its use [1,18]. Despite retrospective studies showing some effectiveness, no randomized controlled trials have confirmed FFP’s efficacy [1,112]. However, FFP is linked to prolonged resolution times and an increased incidence of adverse effects; therefore, it should be reserved for situations where alternative treatments are not available [1,18,110]. In such cases, precautions must be taken to ensure the patient’s airway is protected [1,18,110].

The intravenous (IV) options for treating acute angioedema attacks include plasma-derived C1-INH (pdC1-INH) and recombinant human C1-INH (rhC1-INH) [1,110]. The recommended dosage for pdC1-INH is 20 units/kg rounded up to the nearest vial size [1,110]. Plasma-derived C1-INH is the choice of on-demand treatment for seizures in children, pregnant, and breastfeeding women [18].

RhC1-INH, derived from the milk of transgenic rabbits, offers similar efficacy to pdC1-INH, but it has a shorter half-life, demanding higher doses for acute treatment [1,113]. Nevertheless, it is not used for prophylaxis [1,113]. The recommended dose for rhC1-INH is 50 units/kg, with a maximum of 4200 units per dose [1,110,113]. Both pdC1-INH and rhC1-INH can be used in children aged five and older [1,110]. RhC1-INH is contraindicated for those with a rabbit allergy [1,110].

Icatibant, a bradykinin B2-receptor antagonist, is approved for use in individuals aged 18 and older in the United States [1,110]. Administered via subcutaneous injection, icatibant’s dosage is weight-based, with a maximum of three doses allowed within 24 h if symptoms do not improve within 6 h [110]. Caution is advised when using icatibant in patients with angina or coronary artery disease, as it may reduce coronary blood flow [114].

Ecallantide is another subcutaneous option [1,110]. It a recombinant plasma kallikrein inhibitor that blocks bradykinin production [1,110]. Approved for use in those aged 12 and older, ecallantide is associated with the risk of anaphylaxis and allergic reactions, so it must be administered in a healthcare setting equipped to manage these complications. [115]. The standard adult dose is 30 mg delivered in three separate injections [115].

Laryngeal attacks significantly increase mortality risk, so emergency care is critical for patients experiencing swelling in the larynx, throat, or tongue [1,39]. In cases of respiratory distress, elective intubation should be considered to secure the airway [1,39]. If intubation fails, an emergency cricothyrotomy may be necessary [1,39].

Gastrointestinal attacks typically resolve without treatment, though on-demand treatment, rehydration, and symptom management with antiemetics, antidiarrheal agents, or constipation medications may be needed [1,39].

Primary care providers should educate patients on how to avoid triggers and evaluate the option of long-term prophylaxis [105,108]. Additionally, teaching patients self-treatment techniques is crucial for enhancing their independence and overall quality of life [105,108]. Regular follow-up is essential to minimize morbidity and ensure that treatment remains effective [105,108].

### 3.9. Prophylaxis

#### 3.9.1. Short-Term Prophylaxis

Short-term prophylaxis effectively reduces attack frequency in at-risk individuals [1,116]. It is particularly important for patients undergoing invasive procedures or experiencing trauma and stress [1]. For those on long-term prophylaxis, dosages can be adjusted to provide an IV or subcutaneous dose immediately before an event [1,18]. Alternatively, a 5-day androgen course may be initiated before and continued for a few days after the procedure [1,110].

While pdC1-INH should be administered 1 to 12 h before a procedure, ecallantide and icatibant are unsuitable for prevention due to their short half-life [1,110]. Tranexamic acid (TXA) can be used both acutely and prophylactically in HAE treatment, despite some debate over its efficacy [1,110,117]. TXA may help treat ACE inhibitor-induced angioedema by displacing plasmin to prevent clot breakdown and reduce bradykinin levels and offers benefits for short-term prophylaxis [1,110,117].

#### 3.9.2. Long-Term Prophylaxis

The decision to initiate long-term prophylaxis for HAE is primarily influenced by several factors, including the severity and frequency of attacks, their impact on quality of life, treatment accessibility, and comorbidities [1,110]. First-line prophylactic therapies include the monoclonal plasma kallikrein inhibitor Lanadelumab, intravenous pdC1-INH (Cinryze), and subcutaneous pdC1-INH (Haegarda) [1,110] (Table 5).

Cinryze, approved for both adults and children, is administered every 3 to 4 days, with dosages of 2500 or 1000 units depending on age [1,110] (Table 5). Considerations for its use include the necessity for long-term IV access and the associated risks of infection and vein damage [1,110,112,118,119,120]. Haegarda offers a similar dosing regimen with lower risks related to IV access [1,119,120]. PdC1-INH is preferred for acute treatment and prevention in pregnant and lactating patients [1,119,120,121].

Lanadelumab, approved by the FDA in 2020 for those aged 12 and older, provides long-term prophylaxis with a dosing frequency of once or twice monthly [1,110,122] (Table 5). The initial dose is 300 mg subcutaneously every two weeks, adjustable after 6 months based on attack control [1,123]. Berotralstat, a recently approved oral plasma kallikrein inhibitor, is typically dosed at 150 mg daily, though caution is advised due to potential QT prolongation [1,123].

Second-line options may include anabolic androgens and antifibrinolytics [1] (Table 5). Danazol, an oral option, is effective but associated with significant side effects and is recommended at the lowest effective dose, which may range from 50 to 200 mg daily or every other day [110,124]. The following two dosing strategies are suggested: starting with a high dose (400 or 600 mg daily) and tapering based on tolerability for those needing rapid attack control or starting with a low dose (100 mg daily) and gradually increasing for those not requiring immediate relief [110,124]. Tranexamic acid is an alternative, especially for pregnant patients and children, dosed at 500 mg orally two to three times daily, with a maximum tolerated dose of 3 g [125,126,127] (Table 5). Monitoring for thrombosis is crucial during its use [125,126,127].

## 4. Conclusions

HAE presents a complex challenge requiring a multifaceted approach to management. Advances in understanding the pathophysiology of C1 inhibitor deficiency have led to significant progress in treatment options. Biomarkers have the potential to improve diagnostic accuracy, facilitate personalized management, and support clinical trials in HAE. The advent of novel high-throughput techniques now enables non-targeted multi-omics analyses, allowing for the identification of a cohort of biomarkers for further in-depth research.

The choice of therapy should be guided by factors including the frequency and severity of attacks, patient-specific considerations, and the potential for side effects. The development of targeted therapies, such as plasma-derived and recombinant C1 inhibitor concentrates, bradykinin receptor antagonists, and plasma kallikrein inhibitors, has revolutionized both acute and prophylactic care for HAE patients. Ongoing research continues to expand our options, with emerging therapies promising further advancements in the management of HAE.

Short-term prophylaxis effectively mitigates the risk of attacks during high-risk situations, while long-term prophylaxis options offer sustained protection against recurrent episodes. Despite the efficacy of these newer treatments, individual patient response and treatment tolerability remain critical factors in therapy selection.

Overall, a personalized approach, combining acute management with appropriate prophylaxis and regular patient education, ensures optimal outcomes. Interdisciplinary collaboration among specialists is essential for effective disease management, improving patient quality of life, and reducing the burden of this challenging condition.

## Figures and Tables

**Table 1:** Symptoms associated with different types of HAE.

Type I HAE	Type II HAE	Type III HAE	Others
Cutaneous swelling, particularly in the abdomen Abdominal pain Laryngeal edema, leading to voice changes and swallowing difficulties	Cutaneous swelling, particularly in the abdomen Abdominal pain Laryngeal edema, leading to voice changes and swallowing difficulties	Predominantly cutaneous swelling and abdominal pain Often associated with hormonal influences, particularly estrogen.	Hand and leg involvement Scrotal and penile edema Labial edema Urinary tract involvement Headaches Visual disturbances (blurred vision, diplopia) Ataxia Painful muscle edema Pleural symptoms with effusion Seizures Hemiparesis

**Table 2:** HAE types.

Type I HAE	Type II HAE	Type III HAE
Low levels of C1 inhibitor esterase Low levels of C4 and C2 Normal C1q levels	Normal or increased C1 inhibitor levels but dysfunctional Low levels of C4 and C2 Normal C1q levels	Normal C1 inhibitor esterase and functional C1-INH levels Normal C4 and C1q levels Genetic mutations

**Table 3:** HAE biomarkers.

Biomarkers	Function	Category	References
AgC1-INH	Regulates complement and contact pathways; deficiency leads to uncontrolled bradykinin production	Established	[10,53,54,55,56,57]
C1-INH-C1(r,s) protease-inhibitor complex	These complex forms when C1-INH neutralizes its primary targets—C1r and C1s proteases—which are components of the complement system	Promising	[53,55,56]
fC1-INH	Evaluates the actual ability of the protein to regulate complement and contact system activation, which is essential in preventing the formation of bradykinin	Established	[47,53,54,58,59,60,61,62,63]
Complement C4	Low levels during an attack; diagnostic utility	Established	[53,54,60,61]
Anti-C1-INH IgM antibody	Binds to C1-INH and reduces its functional activity, contributing to uncontrolled complement activation	Emerging	[64,65,66,67]
MASP	Activates the lectin complement pathway	Emerging	[64,65,66,67]
Bradykinin	Primary mediator of angioedema in HAE	Established	[10,54,57,60,63,68,69]
cHK	Indicates bradykinin activation during acute attacks	Promising	[10,70]
PKa	Cleaves high-molecular-weight kininogen to produce bradykinin	Emerging	[10,70]
FXIIa	Initiates the contact activation pathway, leading to bradykinin release	Emerging	[10,71,72,73]
ACE	Degrades bradykinin; may influence HAE attack frequency	Emerging	[10,74,75,76]
Carboxypeptidase N	Involved in bradykinin degradation	Emerging	[10,74,75,76]
aPTT	Measures coagulation pathway activity	Emerging	[73]
APP	Degrades bradykinin; involved in regulating its levels	Emerging	[10,74]
D-dimers	Elevated D-dimer levels indicate increased fibrinolytic activity	Emerging	[55,77,78]
VE-cadherin	Involved in endothelial integrity and permeability	Promising	[77,79,80,81,82,83,84]
VWF	Plays a role in coagulation; linked to endothelial function	Emerging	[77,79,80,81,82,83,84]
VCAM	Modulates leukocyte adhesion and endothelial function	Emerging	[10,79,80,81,82,83,84]
VEGF	Promotes angiogenesis and vascular permeability	Promising	[85,86,87,88]
PAF-AH	Degrades pro-inflammatory lipids	Promising	[85,86,87,88]
ADMA	Modulator of endothelial function and nitric oxide synthesis	Emerging	[10]
CRP	Acute-phase reactant; indicative of systemic inflammation	Promising	[89,90,91,92]
ESR	Marker of inflammation; elevated in chronic disease	Promising	[89,90,91,92]
WBC	Indicator of immune response and infection	Promising	[89,90,91,92]
G-CSF	Stimulates neutrophil production; linked to inflammation	Emerging	[92,93,94]
ILs	Modulate immune response; IL-6 and IL-1 linked to HAE	Promising	[92,93,94]
NO	Modulates vascular tone; related to endothelial function	Emerging	[10,93,95]
TNF	Pro-inflammatory cytokine; linked to vascular permeability	Promising	[92,93,94]
ANGPTs	Modulate vascular permeability, linked to vascular integrity	Emerging	[10,96,97]

**Table 4:** HAE treatment options.

Treatment Type	Medication	Administration	Indications/Notes
Acute treatment	Plasma-derived C1-INH Recombinant human C1-INH Icatibant Ecallantide	20 units/kg (pdC1-INH) rounded to nearest vial size 50 units/kg (maximum 4200 units) Weight-based, maximum of three doses in 24 h if needed 30 mg in three separate injections	First-line treatment; effective within 60 min. Used in children, pregnant, and lactating women. Shorter half-life; not used for prophylaxis. Contraindicated in patients with a rabbit allergy. Approved for ages 18 and older; subcutaneous injection; caution in patients with coronary artery disease. Approved for ages 12 and older; risk of anaphylaxis; administer in healthcare settings.

**Table 5:** HAE long-term prophylaxis.

Treatment Type	Medication	Administration	Indications/Notes
Long-term prophylaxis	Lanadelumab pdC1-INH (Cinryze) Haegarda Danazol Tranexamic acid	300 mg SC every two weeks, adjustable to every 4 weeks 2500 or 1000 units every 3 to 4 days 60 units/kg SC every 3 to 4 days 50 to 200 mg daily/every other day or high dose tapering 500 mg orally two to three times daily (maximum 3 g)	First-line for long-term management; approved for ages 12 and older. Approved for both children and adults. Consider risks associated with long-term IV access. Alternative to IV treatment; fewer risks associated with administration. Second-line treatment; avoid in prepubertal adolescents and pregnant patients. Used off-label; preferred in pregnant patients and children; monitor thrombosis risk.

## References

[1] Sinnathamby E.S., Issa P.P., Roberts L., Norwood H., Malone K., Vemulapalli H., Ahmadzadeh S., Cornett E.M., Shekoohi S., Kaye A.D. (2023). Hereditary Angioedema: Diagnosis, Clinical Implications, and Pathophysiology. Adv Ther..

[2] Wilkerson R.G., Moellman J.J. (2022). Hereditary Angioedema. Emerg. Med. Clin..

[3] Andrejević S., Korošec P., Šilar M., Košnik M., Mijanović R., Bonači-Nikolić B., Rijavec M. (2015). Hereditary angioedema due to C1 inhibitor deficiency in Serbia: Two novel mutations and evidence of genotype–phenotype association. PLoS ONE.

[4] Nasr I.H., Manson A.L., Al Wahshi H.A., Longhurst H.J. (2016). Optimizing hereditary angioedema management through tailored treatment approaches. Expert. Rev. Clin. Immunol..

[5] Longhurst H.J., Bork K. (2019). Hereditary angioedema: An update on causes, manifestations and treatment. Br. J. Hosp. Med. (Lond.).

[6] Bova M., De Feo G., Parente R., De Pasquale T., Gravante C., Pucci S., Nettis E., Triggiani M. (2018). Hereditary and Acquired Angioedema: Heterogeneity of Pathogenesis and Clinical Phenotypes. Int. Arch. Allergy Immunol..

[7] Santacroce R., D’Andrea G., Maffione A.B., Margaglione M., d’Apolito M. (2021). The Genetics of Hereditary Angioedema: A Review. J. Clin. Med..

[8] Kaplan A.P., Joseph K. (2014). Pathogenic mechanisms of bradykinin mediated diseases: Dysregulation of an innate inflammatory pathway. Adv. Immunol..

[9] Maurer M., Bader M., Bas M., Bossi F., Cicardi M., Cugno M., Howarth P., Kaplan A., Kojda G., Leeb-Lundberg F. (2011). New topics in bradykinin research. Allergy.

[10] Porebski G., Kwitniewski M., Reshef A. (2021). Biomarkers in Hereditary Angioedema. Clin. Rev. Allergy Immunol..

[11] Memon R.J., Tiwari V. (2022). Angioedema. StatPearls.

[12] Moellman J.J., Bernstein J.A., Lindsell C., Banerji A., Busse P.J., Camargo C.A., Collins S.P., Craig T.J., Lumry W.R., Nowak R. (2014). A consensus parameter for the evaluation and management of angioedema in the emergency department. Acad. Emerg. Med..

[13] Zuraw B.L. (2008). Hereditary angioedema. N. Engl. J. Med..

[14] Gábos G., Dobru D., Mihály E., Bara N., Dumitrache C., Popa R., Nădășan V., Moldovan D. (2017). Recurrent ascites: A need to evaluate for hereditary angio-oedema. Lancet.

[15] Keeney S., Halalau A. (2017). Anchoring bias in a case of recurrent abdominal pain. BMJ Case Rep..

[16] Elenburg S.N., Assa’ad A.H., Bernstein J.A., Nanda M. (2014). Clinical features of pediatric hereditary angioedema. J. Allergy Clin. Immunol..

[17] Sarkar A., Nwagwu C., Craig T. (2023). Hereditary Angioedema: A Disease Often Misdiagnosed and Mistreated. Primary Care.

[18] Maurer M., Magerl M., Betschel S., Aberer W., Ansotegui I.J., Aygören-Pürsün E., Banerji A., Bara N.A., Boccon-Gibod I., Bork K. (2022). The international WAO/EAACI guideline for the management of hereditary angioedema-The 2021 revision and update. Allergy.

[19] Ghazi A., Grant J.A. (2013). Hereditary angioedema: Epidemiology, management, and role of icatibant. Biologics.

[20] Cicardi M., Agostoni A. (1996). Hereditary angioedema. N. Engl. J. Med..

[21] Quincke H. (1882). Concerning the acute localized edema of the skin. Monatsh Prakt Derm.

[22] Osler W. (1888). Hereditary angioneurotic edema. Am. J. Med. Sci..

[23] Donaldson V.H., Evans R.R. (1963). A biochemical abnormality in hereditary angioneurotic edema: Absence of serum inhibitor of C’ 1-esterase. Am. J. Med..

[24] Nzeako U.C., Frigas E., Tremaine W.J. (2001). Hereditary angioedema: A broad review for clinicians. Arch. Intern. Med..

[25] Dreskin S., Goldman L., Schafer A.I. (2011). Urticaria and angioedema. Cecil Medicine.

[26] Talavera A., Larraona J.L., Ramos J.L., López T., Maraver A., Arias J., Barrios A. (1995). Hereditary angioedema: An infrequent cause of abdominal pain with ascites. Am. J. Gastroenterol..

[27] Agostoni A., Aygören-Pürsün E., Binkley K.E., Blanch A., Bork K., Bouillet L., Bucher C., Castaldo A.J., Cicardi M., Davis A.E. (2004). Hereditary and acquired angioedema: Problems and progress: Proceedings of the third C1 esterase inhibitor deficiency workshop and beyond. J. Allergy Clin. Immunol..

[28] Frank M.M., Gelfand J.A., Atkinson J.P. (1976). Hereditary angioedema: The clinical syndrome and its management. Ann. Intern. Med..

[29] Kesh S., Bernstein J.A. (2022). Isolated angioedema: A review of classification and update on management. Ann. Allergy Asthma Immunol..

[30] Longhurst H.J., Zanichelli A., Caballero T., Bouillet L., Aberer W., Maurer M., Fain O., Fabien V., Andresen I., IOS Study Group (2017). Comparing acquired angioedema with hereditary angioedema (types I/II): Findings from the Icatibant Outcome Survey. Clin. Exp. Immunol..

[31] Levi M., Cohn D.M. (2019). The Role of Complement in Hereditary Angioedema. Transfus. Med. Rev..

[32] Caccia S., Suffritti C., Cicardi M. (2014). Pathophysiology of hereditary angioedema. Pediatr. Allergy Immunol. Pulmonol..

[33] Betschel S.D., Banerji A., Busse P.J., Cohn D.M., Magerl M. (2023). Hereditary Angioedema: A Review of the Current and Evolving Treatment Landscape. The journal of allergy and clinical immunology. In Practice.

[34] Bork K., Wulff K., Steinmüller-Magin L., Braenne I., Staubach-Renz P., Witzke G., Hardt J. (2018). Hereditary angioedema with a mutation in the plasminogen gene. Allergy.

[35] Fijen L.M., Bork K., Cohn D.M. (2021). Current and Prospective Targets of Pharmacologic Treatment of Hereditary Angioedema Types 1 and 2. Clinic. Rev. Allerg. Immunol..

[36] Bernstein J.A. (2018). Severity of hereditary angioedema, prevalence, and diagnostic considerations. Am. J. Manag. Care.

[37] Henao M.P., Kraschnewski J.L., Kelbel T., Craig T.J. (2016). Diagnosis and screening of patients with hereditary angioedema in primary care. Ther. Clin. Risk. Manag..

[38] Iwanami K., Okano T., Ohara O., Morio T. (2019). Recurrent acute abdomen as the main manifestation of hereditary angioedema. Intern. Med..

[39] Bork K., Hardt J., Witzke G. (2012). Fatal laryngeal attacks and mortality in hereditary angioedema due to C1-INH deficiency. J. Allergy Clin. Immunol..

[40] Jindal A.K., Bishnoi A., Dogra S. (2021). Hereditary angioedema: Diagnostic algorithm and current treatment concepts. Indian Dermatol Online J..

[41] Jacobs J., Neeno T. (2021). The importance of recognizing and managing a rare form of angioedema: Hereditary angioedema due to C1-inhibitor deficiency. Postgrad. Med..

[42] Caballero T., Baeza M.L., Cabañas R., Campos A., Cimbollek S., Gómez-Traseira C., González-Quevedo T., Guilarte M., Jurado-Palomo G.J., Larco J.I. (2011). Consensus statement on the diagnosis, management, and treatment of angioedema mediated by bradykinin. Part I. Classification, epidemiology, pathophysiology, genetics, clinical symptoms, and diagnosis. J. Investig. Allergol. Clin. Immunol..

[43] Cicardi M., Zanichelli A. (2013). Diagnosing angioedema. Immunol. Allergy Clin. N. Am..

[44] Nowicki R.J., Grubska-Suchanek E., Porêbski G., Kowalski M.L., Jahnz-Różyk K., Matuszewski T., Rudnicka L., Kulus M., Barañska-Rybak W., Czajkowski R. (2020). Angioedema. Interdisciplinary diagnostic and therapeutic recommendations of the Polish Dermatological Society (PTD) and Polish Society of Allergology (PTA). Postepy Dermatol. Alergol..

[45] Karim Y., Griffiths H., Deacock S. (2004). Normal complement C4 values do not exclude hereditary angioedema. J. Clin. Pathol..

[46] Tarzi M.D., Hickey A., Forster T., Mohammadi M., Longhurst H.J. (2007). An evaluation of tests used for the diagnosis and monitoring of C1 inhibitor deficiency: Normal serum C4 does not exclude hereditary angio-oedema. Clin. Exp. Immunol..

[47] Wagenaar-Bos I.G., Drouet C., Aygoren-Pursun E., Bork K., Bucher C., Bygum A., Farkas H., Fust G., Gregorek H., Hack C.E. (2008). Functional C1-inhibitor diagnostics in hereditary angioedema: Assay evaluation and recommendations. J. Immunol. Methods.

[48] Aabom A., Bygum A., Koch C. (2017). Complement factor C4 activation in patients with hereditary angioedema. Clin. Biochem..

[49] Jindal A.K., Reshef A., Longhurst H., GEHM workgroup (Global Equity in HAE Management) (2021). Mitigating disparity in health-care resources between countries for management of hereditary angioedema. Clin. Rev. Allergy Immunol..

[50] Johnston D.T. (2011). Diagnosis and management of hereditary angioedema. J. Osteopath. Med..

[51] Germenis A.E., Margaglione M., Pesquero J.B., Farkas H., Cichon S., Csuka D., López Lera A., Rijavec M., Jolles S., Szilagyi A. (2020). International consensus on the use of genetics in the management of hereditary angioedema. J. Allergy Clin. Immunol. Pract..

[52] Veronez C.L., Aabom A., Martin R.P., Filippelli-Silva R., Gonçalves R.F., Nicolicht P., Mendes A.R., Da Silva J., Guilarte M., Grumach A.S. (2019). Genetic Variation of Kallikrein-Kinin System and Related Genes in Patients with Hereditary Angioedema. Front. Med..

[53] Csuka D., Füst G., Farkas H., Varga L. (2011). Parameters of the classical complement pathway predict disease severity in hereditary angioedema. Clin. Immunol..

[54] Germenis A.E., Cicardi M. (2019). Driving towards precision medicine for angioedema without wheals. J. Autoimmun..

[55] Cugno M., Nuijens J., Hack E., Eerenberg A., Frangi D., Agostoni A., Cicardi M. (1990). Plasma levels of C1^−^ inhibitor complexes and cleaved C1^−^ inhibitor in patients with hereditary angioneurotic edema. J. Clin. Investig..

[56] Cugno M., Hack C.E., de Boer J.P., Eerenberg A.J., Agostoni A., Cicardi M. (1993). Generation of plasmin during acute attacks of hereditary angioedema. J. Lab. Clin. Med..

[57] Cicardi M., Bergamaschini L., Cugno M., Hack E., Agostoni G., Agostoni A. (1991). Long-term treatment of hereditary angioedema with attenuated androgens: A survey of a 13-year experience. J. Allergy Clin. Immunol..

[58] Li H.H., Busse P., Lumry W.R., Frazer-Abel A., Levy H., Steele T., Dayno J., Riedl M. (2015). Comparison of chromogenic and ELISA functional C1 inhibitor tests in diagnosing hereditary angioedema. J. Allergy Clin. Immunol. Pract..

[59] Gompels M.M., Lock R.J., Morgan J.E., Osborne J., Brown A., Virgo P.F. (2002). A multicentre evaluation of the diagnostic efficiency of serological investigations for C1 inhibitor deficiency. J. Clin. Pathol..

[60] Kelemen Z., Moldovan D., Mihály E., Visy B., Széplaki G., Csuka D., Füst G., Farkas H., Varga L. (2010). Baseline level of functional C1-inhibitor correlates with disease severity scores in hereditary angioedema. Clin. Immunol..

[61] Longhurst H., Cicardi M., Craig T., Bork K., Grattan C., Baker J., Li H.H., Reshef A., Bonner J., Bernstein J.A. (2017). Prevention of hereditary angioedema attacks with a subcutaneous C1 inhibitor. N. Engl. J. Med..

[62] Hack C.E., Relan A., van Amersfoort E.S., Cicardi M. (2012). Target levels of functional C1-inhibitor in hereditary angioedema. Allergy.

[63] Kaplan A.P., Pawaskar D., Chiao J. (2020). C1 inhibitor activity and angioedema attacks in patients with hereditary angioedema. J. Allergy Clin. Immunol. Pract..

[64] Betschel S., Badiou J., Binkley K., Borici-Mazi R., Hébert J., Kanani A., Keith P., Lacuesta G., Waserman S., Yang B. (2019). The International/Canadian Hereditary Angioedema Guideline. Allergy Asthma Clin. Immunol..

[65] Varga L., Széplaki G., Visy B., Füst G., Harmat G., Miklós K., Németh J., Cervenak L., Karádi I., Farkas H. (2007). C1-inhibitor (C1-INH) autoantibodies in hereditary angioedema. Strong correlation with the severity of disease in C1-INH concentrate naïve patients. Mol. Immunol..

[66] Hansen C.B., Csuka D., Munthe-Fog L., Varga L., Farkas H., Hansen K.M., Koch C., Skjødt K., Garred P., Skjoedt M.O. (2015). The levels of the lectin pathway serine protease MASP-1 and its complex formation with C1 inhibitor are linked to the severity of hereditary angioedema. J. Immunol..

[67] Csuka D., Munthe-Fog L., Hein E., Zotter Z., Prohászka Z., Farkas H., Varga L., Garred P. (2014). Activation of the ficolin-lectin pathway during attacks of hereditary angioedema. J. Allergy Clin. Immunol..

[68] Nussberger J., Cugno M., Amstutz C., Cicardi M., Pellacani A., Agostoni A. (1998). Plasma bradykinin in angio-oedema. Lancet.

[69] Nussberger J., Cugno M., Cicardi M., Agostoni A. (1999). Local bradykinin generation in hereditary angioedema. J. Allergy Clin. Immunol..

[70] Suffritti C., Zanichelli A., Maggioni L., Bonanni E., Cugno M., Cicardi M. (2014). High-molecular-weight kininogen cleavage correlates with disease states in the bradykinin-mediated angioedema due to hereditary C1-inhibitor deficiency. Clin. Exp. Allergy.

[71] Cugno M., Cicardi M., Coppola R., Agostoni A. (1996). Activation of the factor XII and cleavage of high molecular weight kininogen during acute attacks in hereditary and acquired C1-inhibitor deficiencies. Immunopharmacology.

[72] Joseph K., Tuscano T.B., Kaplan A.P. (2008). Studies of the mechanisms of bradykinin generation in hereditary angioedema plasma. Ann. Allergy Asthma Immunol..

[73] Csuka D., Veszeli N., Imreh É., Zotter Z., Skopál J., Prohászka Z., Varga L., Farkas H. (2015). Comprehensive study into the activation of the plasma enzyme systems during attacks of hereditary angioedema due to C1-inhibitor deficiency. Orphanet. J. Rare Dis..

[74] Dessart P., Defendi F., Humeau H., Nicolie B., Sarre M.E., Charignon D., Ponard D., Cichon S., Drouet C., Martin L. (2015). Distinct conditions support a novel classification for bradykinin-mediated angio-oedema. Dermatology.

[75] Charignon D., Ghannam A., Defendi F., Ponard D., Monnier N., López Trascasa M., Launay D., Caballero T., Djenouhat K., Fain O. (2014). Hereditary angioedema with F12 mutation: Factors modifying the clinical phenotype. Allergy.

[76] Defendi F., Charignon D., Ghannam A., Baroso R., Csopaki F., Allegret-Cadet M., Ponard D., Favier B., Cichon S., Nicolie B. (2013). Enzymatic assays for the diagnosis of bradykinin-dependent angioedema. PLoS ONE.

[77] Cugno M., Zanichelli A., Bellatorre A.G., Griffini S., Cicardi M. (2009). Plasma biomarkers of acute attacks in patients with angioedema due to C1-inhibitor deficiency. Allergy.

[78] Reshef A., Zanichelli A., Longhurst H., Relan A., Hack C.E. (2015). Elevated D-dimers in attacks of hereditary angioedema are not associated with increased thrombotic risk. Allergy.

[79] Bouillet L., Mannic T., Arboleas M., Subileau M., Massot C., Drouet C., Huber P., Vilgrain I. (2011). Hereditary angioedema: Key role for kallikrein and bradykinin in vascular endothelial-cadherin cleavage and edema formation. J. Allergy Clin Immunol..

[80] Kajdácsi E., Jani P.K., Csuka D., Varga L.Á., Prohászka Z., Farkas H., Cervenak L. (2014). Endothelial cell activation during edematous attacks of hereditary angioedema types I and II. J. Allergy Clin. Immunol..

[81] Czúcz J., Schaffer G., Csuka D., Walentin S., Kunde J., Prohászka Z., Farkas H., Cervenak L. (2012). Endothelial cell function in patients with hereditary angioedema: Elevated soluble E-selectin level during inter-attack periods. J. Clin. Immunol..

[82] Kajdácsi E., Jani P.K., Csuka D., Varga L., Prohászka Z., Farkas H., Cervenak L. (2016). Novel vasoregulatory aspects of hereditary angioedema: The role of arginine vasopressin, adrenomedullin and endothelin-1. J. Clin. Immunol..

[83] Kajdácsi E., Varga L., Prohászka Z., Farkas H., Cervenak L. (2016). Atrial natriuretic peptide as a novel biomarker of hereditary angioedema. Clin. Immunol..

[84] Demirturk M., Akpinar T.S., Kose M., Gelincik A., Colakoğlu B., Buyukozturk S. (2017). Endocan: A novel marker of endothelial dysfunction in C1-inhibitor-deficient hereditary angioedema. Int. Arch. Allergy Immunol..

[85] Loffredo S., Bova M., Suffritti C., Borriello F., Zanichelli A., Petraroli A., Varricchi G., Triggiani M., Cicardi M., Marone G. (2016). Elevated plasma levels of vascular permeability factors in C1 inhibitor-deficient hereditary angioedema. Allergy.

[86] Loffredo S., Ferrara A.L., Bova M., Borriello F., Suffritti C., Veszeli N., Petraroli A., Galdiero M.R., Varricchi G., Granata F. (2018). Secreted phospholipases A2 in hereditary angioedema with C1-inhibitor deficiency. Front. Immunol..

[87] Bova M., Suffritti C., Bafunno V., Loffredo S., Cordisco G., Del Giacco S., De Pasquale T.M.A., Firinu D., Margaglione M., Montinaro V. (2020). Impaired control of the contact system in hereditary angioedema with normal C1-inhibitor. Allergy.

[88] Ferrara A.L., Bova M., Petraroli A., Veszeli N., Galdiero M.R., Braile M., Marone G., Cristinziano L., Marcella S., Modestino L. (2020). Hereditary angioedema attack: What happens to vasoactive mediators?. Int. Immunopharmacol..

[89] Márkus B., Veszeli N., Temesszentandrási G., Farkas H., Kalabay L. (2019). Serum fetuin-A, tumor necrosis factor alpha and C-reactive protein concentrations in patients with hereditary angioedema with C1-inhibitor deficiency. Orphanet. J. Rare Dis..

[90] Veszeli N., Csuka D., Zotter Z., Imreh É., Józsi M., Benedek S., Varga L., Farkas H. (2015). Neutrophil activation during attacks in patients with hereditary angioedema due to C1-inhibitor deficiency. Orphanet. J. Rare Dis..

[91] Arcoleo F., Salemi M., la Porta A., Selvaggio V., Mandalà V., Muggeo V., Misiano G., Milano S., Romano G.C., Cillari E. (2014). Upregulation of cytokines and IL-17 in patients with hereditary angioedema. Clin. Chem. Lab Med..

[92] Salemi M., Mandalà V., Muggeo V., Misiano G., Milano S., Colonna-Romano G., Arcoleo F., Cillari E. (2016). Growth factors and IL-17 in hereditary angioedema. Clin. Exp. Med..

[93] Hofman Z.L.M., Relan A., Hack C.E. (2014). C-reactive protein levels in hereditary angioedema. Clin. Exp. Immunol..

[94] Food and Drug Administration, HHS (2008). International Conference on Harmonisation; Guidance on E15 Pharmacogenomics Definitions and Sample Coding; Availability. Notice. Fed. Regist..

[95] Demirtürk M., Gelincik A., Çinar S., Kilercik M., Onay-Ucar E., Çolakoğlu B., Arda N., Büyüköztürk S., Deniz G. (2014). Increased eNOS levels in hereditary angioedema. Int. Immunopharmacol..

[96] Germenis A.E., Speletas M. (2016). Genetics of hereditary angioedema revisited. Clin. Rev. Allergy Immunol..

[97] Beard N., Frese M., Smertina E., Mere P., Katelaris C., Mills K. (2022). Interventions for the long-term prevention of hereditary angioedema attacks. Cochrane Database Syst. Rev..

[98] Spath P.J., Wuthrich B., Butler R. (1984). Quantification of C1-inhibitor functional activities by immunodiffusion assay in plasma of patients with hereditary angioedema—evidence of a functionally critical level of C1- inhibitor concentration. Complement.

[99] Cichon S., Martin L., Hennies H.C., Müller F., Van Driessche K., Karpushova A., Stevens W., Colombo R., Renné T., Drouet C. (2006). Increased activity of coagulation factor XII (Hageman Factor) causes hereditary angioedema type III. Am. J. Hum. Genet..

[100] Bork K., Kleist R., Hardt J., Witzke G. (2009). Kallikrein-kinin system and fibrinolysis in hereditary angioedema due to factor XII gene mutation Thr309Lys. Blood Coagul. Fibrinolysis.

[101] Konings J., Cugno M., Suffritti C., Ten Cate H., Cicardi M., Govers-Riemslag J.W. (2013). Ongoing contact activation in patients with hereditary angioedema. PLoS ONE.

[102] Marlu R., Deroux A., Du-Thanh A., Boccon-Gibod I., Launay D., Bouillet L. (2017). Normal PAI-2 level in French FXII-HAE patients. J. Allergy Clin. Immunol..

[103] Bas M., Storck K., Strassen U. (2017). Potential biomarkers for the diagnosis of angiotensin-converting enzyme inhibitor-induced angioedema. ORL J. Otorhinolaryngol. Relat. Spec..

[104] Caballero T. (2021). Treatment of Hereditary Angioedema. J. Investig. Allergol. Clin. Immunol..

[105] Agboola F., Lubinga S., Carlson J., Lin G.A., Dreitlein W.B., Pearson S.D. (2019). The Effectiveness and Value of Lanadelumab and C1 Esterase Inhibitors for Prophylaxis of Hereditary Angioedema Attacks. J. Manag. Care Spec. Pharm..

[106] Riedl M.A., Banerji A., Manning M.E., Burrell E., Joshi N., Patel D., Machnig T., Tai M.H., Watson D.J. (2018). Treatment patterns and healthcare resource utilization among patients with hereditary angioedema in the United States. Orphanet. J. Rare Dis..

[107] Cardarelli W.J. (2018). Economic burden limiting proper healthcare delivery, management, and improvement of patient outcomes. Am. J. Manag. Care.

[108] Murphy E., Donahue C., Omert L., Persons S., Tyma T.J., Chiao J., Lumry W. (2019). Training patients for self-administration of a new subcutaneous C1-inhibitor concentrate for hereditary angioedema. Nurs. Open.

[109] Busse P.J., Christiansen S.C., Riedl M.A., Banerji A., Bernstein J.A., Castaldo A.J., Craig T., Davis-Lorton M., Frank M.M., Li H.H. (2021). US HAEA medical advisory board 2020 guidelines for the management of hereditary angioedema. J. Allergy Clin. Immunol. Pract..

[110] Wentzel N., Panieri A., Ayazi M., Ntshalintshali S.D., Pourpak Z., Hawarden D., Potter P., Levin M.E., Fazlollahi M.R., Peter J. (2019). Fresh frozen plasma for on-demand hereditary angioedema treatment in South Africa and Iran. World Allergy Organ. J..

[111] Gompels M.M., Lock R.J., Abinun M. (2005). C1 inhibitor deficiency: Consensus document. Clin. Exp. Immunol..

[112] Gandhi P.K., Gentry W.M., Bottorff M.B. (2012). Thrombotic events associated with C1 esterase inhibitor products in patients with hereditary angioedema: Investigation from the United States Food and Drug Administration adverse event reporting system database. Pharmacother. J. Hum. Pharmacol. Drug Ther..

[113] Deeks E.D. (2010). Icatibant. Drugs.

[114] Craig T.J., Li H.H., Riedl M., Bernstein J.A., Lumry W.R., MacGinnitie A.J., Stolz L.E., Biedenkapp J., Chyung Y. (2015). Characterization of anaphylaxis after ecallantide treatment of hereditary angioedema attacks. J. Allergy Clin. Immunol. Pract..

[115] Magerl M., Frank M., Lumry W., Bernstein J., Busse P., Craig T., Martinez-Saguer I., Riedl M.A., Shapiro R., Edelman J. (2017). Short-term prophylactic use of C1-inhibitor concentrate in hereditary angioedema: Findings from an international patient registry. Ann. Allergy Asthma Immunol..

[116] Horiuchi T., Hide M., Yamashita K., Ohsawa I. (2018). The use of tranexamic acid for on-demand and prophylactic treatment of hereditary angioedema: A systematic review. J. Cutan Immunol. Allergy.

[117] Wang K., Geiger H., McMahon A. (2021). Tranexamic acid for ACE inhibitor induced angioedema. Am. J. Emerg. Med..

[118] Li H.H., Moldovan D., Bernstein J.A., Reshef A., Porebski G., Stobiecki M., Baker J., Levy R., Relan A., Riedl M. (2015). Recombinant human-C1 inhibitor is effective and safe for repeat hereditary angioedema attacks. J. Allergy Clin. Immunol. Pract..

[119] Aygören-Pürsün E., Soteres D., Moldovan D., Christensen J., Van Leerberghe A., Hao J., Schranz J., Jacobson K.W., Martinez-Saguer I. (2017). Preventing hereditary angioedema attacks in children using Cinryze^®^: Interim efficacy and safety phase 3 findings. Int. Arch. Allergy Immunol..

[120] Lumry W.R., Craig T., Zuraw B., Longhurst H., Baker J., Li H.H., Bernstein J.A., Anderson J., Riedl M.A., Manning M.E. (2018). Health-related quality of life with subcutaneous C1-inhibitor for prevention of attacks of hereditary angioedema. J. Allergy Clin. Immunol. Pract..

[121] Caballero T., Farkas H., Bouillet L., Bowen T., Gompel A., Fagerberg C., Bjökander J., Bork K., Bygum A., Cicardi M. (2012). International consensus and practical guidelines on the gynecologic and obstetric management of female patients with hereditary angioedema caused by C1 inhibitor deficiency. J. Allergy Clin. Immunol..

[122] Banerji A., Riedl M.A., Bernstein J.A., Cicardi M., Longhurst H.J., Zuraw B.L., Busse P.J., Anderson J., Magerl M., Martinez-Saguer I. (2018). Effect of lanadelumab compared with placebo on prevention of hereditary angioedema attacks: A randomized clinical trial. JAMA.

[123] Hwang J.R., Hwang G., Johri A., Craig T. (2019). Oral plasma kallikrein inhibitor BCX7353 for treatment of hereditary angioedema. Immunotherapy.

[124] Zuraw B.L., Davis D.K., Castaldo A.J., Christiansen S.C. (2016). Tolerability and effectiveness of 17-α-alkylated androgen therapy for hereditary angioedema: A re-examination. J. Allergy Clin. Immunol. Pract..

[125] Wintenberger C., Boccon-Gibod I., Launay D., Fain O., Kanny G., Jeandel P.Y., Martin L., Gompel A., Bouillet L. (2014). Tranexamic acid as maintenance treatment for non-histaminergic angioedema: Analysis of efficacy and safety in 37 patients. Clin. Exp. Immunol..

[126] Henry Li H., Riedl M., Kashkin J. (2019). Update on the Use of C1-Esterase Inhibitor Replacement Therapy in the Acute and Prophylactic Treatment of Hereditary Angioedema. Clin. Rev. Allergy Immunol..

[127] Greve J., Strassen U., Gorczyza M., Dominas N., Frahm U.M., Mühlberg H., Wiednig M., Zampeli V., Magerl M. (2016). Prophylaxis in hereditary angioedema (HAE) with C1 inhibitor deficiency. J. Dtsch. Dermatol. Ges..

